# Cost-Effective Strategies for Mitigating a Future Influenza Pandemic with H1N1 2009 Characteristics

**DOI:** 10.1371/journal.pone.0022087

**Published:** 2011-07-08

**Authors:** Nilimesh Halder, Joel K. Kelso, George J. Milne

**Affiliations:** School of Computer Science and Software Engineering, University of Western Australia, Crawley, Western Australia, Australia; Center for Complex Networks and Systems Research, Indiana University at Bloomington, United States of America

## Abstract

**Background:**

We performed an analysis of the cost-effectiveness of pandemic intervention strategies using a detailed, individual-based simulation model of a community in Australia together with health outcome data of infected individuals gathered during 2009–2010. The aim was to examine the cost-effectiveness of a range of interventions to determine the most cost-effective strategies suitable for a future pandemic with H1N1 2009 characteristics.

**Methodology/Principal Findings:**

Using transmissibility, age-stratified attack rates and health outcomes determined from H1N1 2009 data, we determined that the *most cost-effective* strategies involved treatment and household prophylaxis using antiviral drugs combined with *limited duration* school closure, with costs ranging from $632 to $777 per case prevented. When school closure was used as a sole intervention we found the use of limited duration school closure to be significantly more cost-effective compared to continuous school closure, a result with applicability to countries with limited access to antiviral drugs. Other social distancing strategies, such as reduced workplace attendance, were found to be costly due to productivity losses.

**Conclusion:**

The mild severity (low hospitalisation and case fatality rates) and low transmissibility of H1N1 2009 meant that health treatment costs were dominated by the higher productivity losses arising from workplace absence due to illness and childcare requirements following school closure. Further analysis for higher transmissibility but with the same, mild severity had no effect on the overall findings.

## Introduction

The 2009 influenza A/H1N1 pandemic has provided a unique opportunity to examine the effectiveness of a range of interventions used to lessen the number of those becoming infected, the attack rate, using data collected during the pandemic. High quality data collected during the period 2009–2010 has been used to populate a detailed demographic and mobility simulation model of an actual community of ∼30,000 persons in Australia, creating a model with high realism. In contrast, previous modelling studies used to determine the effectiveness of pandemic interventions have relied on post-pandemic estimations of the defining characteristics of the pandemic. Data collected during the recent pandemic include the reproduction number [1], [2], [3], [4], [5], serial interval [1], [2], [5], age-specific attack rate profile [6] and health outcomes of those infected, such as hospitalisation, intensive care and mortality rates. The resulting models [7], [8], [9], [10], [11] have been used to determine the effectiveness (in terms of attack rate reduction) of interventions used in the period 2009-2010 together with examination of other strategies which may be more effective. In the case of this study we have used an individual-based simulation model [12], [13], [14] together with health outcome data on influenza patients in Western Australia during the pandemic period to determine the cost-effectiveness of antiviral drug and social distancing interventions. We have considered interventions actually used in 2009-2010 together with other (combined) intervention strategies to determine which are the most cost-effective for a pandemic with H1N1 2009 characteristics. These results are available to inform public health authorities as to which intervention strategies are cost-optimal.

Antiviral drugs and school closure are considered as frontline pandemic mitigation strategies to reduce the illness attack rate to a low level, either to prevent an epidemic or to buy time for a vaccine to be developed and distributed. Other social distancing strategies such as community gathering and work place attendance reduction are also recommended to control infection spread [15], [16], [17], [18]. The 2009 pandemic provides a unique opportunity for public health authorities to practically review their pandemic mitigation guidelines in the light of the limited success in containing and controlling the pandemic. In some countries there was hesitancy in the use of antiviral drugs; for example in Australia, with one of the largest per capita antiviral drug stockpiles prior to the 2009 pandemic, there was not a rapid and comprehensive use of antiviral drugs for treatment and prophylaxis. A number of studies [7], [8], [9], [10], [11] have reviewed the effectiveness of the 2009 interventions (in terms of reduction in attack rate) and some have suggested more effective strategies than those used. However to determine which strategies are optimal it is important to also determine which of the effective strategies are also cost-effective.

In this study we have used actual hospitalisation, ICU treatment and mortality data from influenza cases in Western Australia in 2009/2010 as the measure of pandemic severity, and used this data to determine the total costs involved. We have evaluated the cost-effectiveness of a wide spectrum of intervention strategies, including limited duration school closure and a range of antiviral strategies, singly and in combination. This comprehensive economic analysis has allowed us to determine the most cost-effective strategies applicable to a future influenza pandemic with H1N1 2009 characteristics. As these characteristics relate closely to seasonal influenza, with similar reproduction numbers of ∼1.3 and similar severity in terms of case fatality rates, these results are applicable more generally.

## Methods

**Figure 1:**
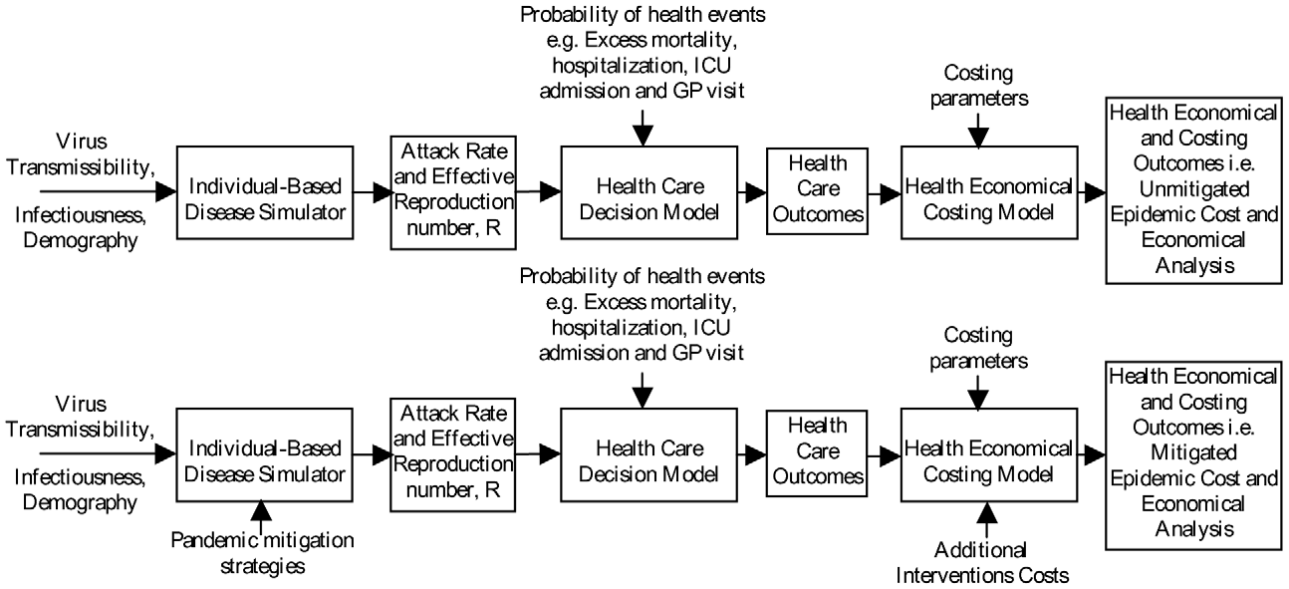
Schematic presentation of methodology.

We used a detailed, individual-based simulation model of a real community in the south-west of Western Australia (Albany) with a population of approximately 30,000 to simulate the dynamics of a pandemic with an illness attack rate and effective reproduction number (R) similar to that of influenza A/H1N1 2009. Comparing simulations with and without interventions in place allowed us to determine the effect which a range of interventions have on reducing the attack rate and on the health profile of each individual in the modelled community. The outcomes of the simulation model were then used by a health-care decision process to determine health-care outcomes (hospitalisation, ICU treatment and deaths) based on data collected during the H1N1 2009 pandemic. These health-care outcomes, together with cost data drawn from the literature, were applied to a costing process, as shown in Figure 1. These analyses determine the baseline (unmitigated, i.e. without intervention) and mitigated (with intervention) pandemic costs and thus permit us to determine the cost-effectiveness of a range of intervention strategies, including those used during the H1N1 2009 influenza pandemic. Furthermore, they give guidance as to which intervention strategies are the most cost-effective in terms of cost per case avoided due to intervention usage.

### Simulation Model

Our individual-based simulation model had been developed by using census, state and local government data to construct a human contact network involving households, schools, childcare centres, workplaces and a regional hospital. Census data was used to populate each household in our modelled community with exact numbers of individuals, with their ages in one of 7 age classes. These ages are used to allocate children to appropriate schools and classes, and adults to workplaces using data on workplaces and schools in the community. The age of individuals is also used when modelling the disease profile in individuals where we use age differential attack rate data for the 2009 pandemic, obtained from the Western Australia Department of Health (personal communication with Dr Gary Dowse).

The modelled community is represented as a dynamic contact network which changes the spatial locations of individuals through time. Individuals move from their households to schools, workplaces and into the wider community during the day, returning to home in the evening. The simulation mechanism underpinning the model captures this changing contact pattern twice per day with each day divided into 12 hour day/night cycles. During each cycle the nominal location of every person was determined and individuals occupying the same location were assumed to come into potential infective contact. In addition, community-wide interaction was modelled by assuming that active individuals would contact other active individuals each day, with contact being random but biased towards contact between people with nearby home locations. We assumed that an average of one new infection per day was stochastically introduced into the population during the whole period of the simulations. This model was previously developed to determine the effectiveness of social distancing and vaccination measures for a possible future H5N1 pandemic [12], [13], [14] and was subsequently used to examine antiviral and school closure interventions which were employed in the H1N1 2009 influenza pandemic [7], [8], [9].

We have further refined this model to reflect the biology of the A/H1N1 2009 influenza virus strain according to information available in 2010 [5]. Transmission of infection between infectious and susceptible individuals who came into infective contact was resolved stochastically. The probability of transmission was calculated as a function of the state of the infectious (I_i_) and susceptible (I_s_) individuals involved at the time of contact, as given by:
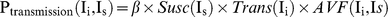



Each factor contributing to the transmission probability is described in [7], [8], [9]. The basic transmission probability (β), capturing the infectivity of the virus strain, was chosen to give an unmitigated epidemic with an effective reproduction number R of 1.2 [5]. We examined a wide range of intervention strategies such as school closure, antiviral drugs treatment and prophylaxis, workplace non-attendance (WP – a 50% reduction in workplace attendance) and community contact reduction (CCR – a 50% reduction in community contact) both individually and in combination. Some of the intervention strategies were applied during the H1N1 2009 influenza pandemic in various countries, while some are examined to determine their potential cost-effectiveness when used in containing and controlling a future influenza pandemic. A detailed description of each modelled intervention strategy is given in [7], [8], [9], [13].

### Economic Model

In this study we used a health-care decision process to determine the health outcomes (such as a family physician (GP) visit, hospitalisation, ICU treatment and death: see Tables 1 and 2) of the total modelled community using medical data related to H1N1 cases during the 2009 pandemic. This model took the outcomes of our individual-based simulation model, which capture the infective profile of each individual in the community; that is, who was infected, when and by whom. As each individual is in one of 7 age classes, age-specific probabilities which relate to each possible health outcome (such as those listed above) of those infected are also taken as input. These age-specific probabilities were estimated using data from the Department of Health, Western Australia (personal communication with Dr. Gary Dowse). The output of the health-care decision process thus defines the severity of a pandemic in terms of the proportion of the population needing medical attention, the proportion being hospitalised, the rate of ICU admission and the mortality rate.

**Table 1:** Age-stratified health-care decision model and cost analysis parameters.

Parameter name	Age Groups				Source
	0–5	6–17	18–64	65+	
P(M|S)^1^	0.013	0.036	0.031	0.002	Calculated from WA Health Data
P(H|S)^1^	0.0006	0.002	0.002	0.00009	Calculated from WA Health Data
P(I|S)^1^	0.00006	0.0002	0.0002	0.000009	Calculated from WA Health Data
P(D|S)^1^	0.00002	0.00006	0.00005	0.000003	Calculated from WA Health Data
Average life-expectancy (years)	76.16	67.88	39.7	14.9	[23]
Average hospital stay (days)	4 days	4 days	4 days	4 days	[21]
Average ICU stay (days)	7 days	7 days	7 days	7 days	[25]

1 Table shows probability of health care outcome conditional on symptomatic illness (S). Health care outcomes: M – general practitioner visit, H – hospitalization, I – intensive care unit admission, D – death.

**Table 2:** Cost analysis model parameters.

Cost analysis assumptions	Values in US$	Source
Average wages (per week)	$836	[23]
Average cost for school closure (per day per student)	$19.22	[22]
Average GP visit cost	$106.97	[20]
Average hospitalization cost (per day)	$1042	[20]
Average ICU stay cost (per day)	$2084	assumed
Antiviral cost per course	$24.81	[20]
Antiviral dispensing cost per course	$31.22	[20]

We focused on determining the total economic cost to society incurred during an influenza pandemic. Total costs involve both direct health-care costs (e.g. the cost of medical attention due to a GP visit, or for hospitalisation) and costs due to productivity loss. Productivity losses arise from pandemic related deaths and illness together with those due to interventions such as (partial) workplace closure and child-care of an ill child. Pharmaceutical costs (e.g. costs related to antiviral drugs) are also estimated. Productivity losses due to death were discounted at 3% annually (which is a standard discount used to express all future income as a present-day value) and all costs are reported in 2010 US dollars using the consumer price index adjustments [19].

In our baseline unmitigated pandemic scenario the total hospitalisation cost is measured by summing age-specific hospitalisation costs. We estimated age-specific hospitalisation costs by multiplying the average cost per day by average length of stay for each age group. We assumed an average hospitalisation cost of $1042 US dollar per day [20], [21]. We also calculated costs for intensive care unit (ICU) treatment using the same method. The costs of treatment and length of stay in hospital (both ICU and non-ICU) which are used in establishing the overall cost of unmitigated and intervention mitigated epidemics in the modelled community are given in Tables 1 and 2. We further estimated the consultation fee of a general practitioner (GP) physician visit for each influenza case at $106.97 [20]. Note that all costs are based on US sources and given in 2010 US dollars.

Productivity loss due to death was calculated from the net present value of future earnings for an averaged-aged person in each age group. It was estimated by multiplying age-specific death in each age group by average earning expectancy in years and by average annual income. We also estimated productivity losses due to illness and interventions (e.g. due to child-care resulting from school closure, workplace non-attendance etc.) multiplying average wages by work-days lost due to illness and interventions. The number of work-days lost were determined from the day-to-day outbreak data generated by our individual-based simulation model. We estimated the cost of school-days lost due to school closure interventions by multiplying average daily cost in a school per student by the number of school-days lost obtained from the simulator. The assumed values of a weekly wage of a working person and the daily cost per student in a school were $836 and $19.22 [22], [23] respectively; these school costs cover additional teaching needed to “make up” missed classes due to absence.

In our analysis we assumed pharmaceutical costs of $24.81 per antiviral course together with administration and dispensing costs of $31.22 per course [20]. We used 2010 US dollar values in determining total costs to make our results readily convertible to a wide range of countries. All costs are based on US sources. In the discussion section we comment on alternative sources of health care and economic cost data.

## Results

**Figure 2:**
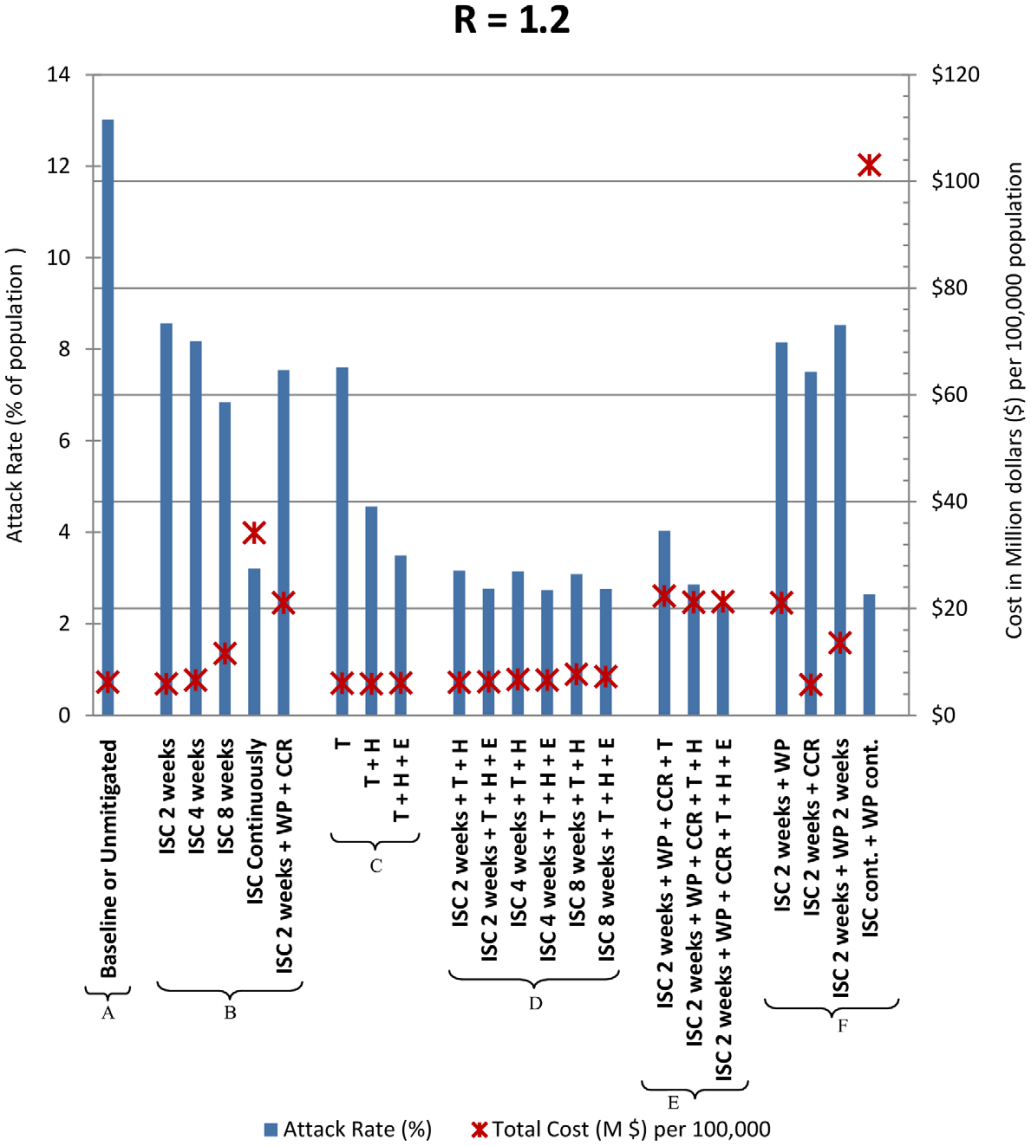
Cost-effectiveness of intervention strategies. Cost effectiveness is shown in terms of illness attack rate (%) and cost in million dollars per 100,000 population. Intervention strategies are abbreviated as follows: ISC – individual school closure, T – antiviral treatment, H – household antiviral prophylaxis, E – extended antiviral prophylaxis, WP – 50% workplace closure (4 weeks if no duration is stated), CCR – 50% community contact reduction. “cont.” refers to continuous school or workplace closure.

The main outcomes of our study are shown in Figure 2 and Table 3 as an average of 40 realizations of each epidemic that have been simulated using our individual-based simulation model. The total cost of an unmitigated (baseline) pandemic and each intervention mitigated pandemic scenario have been described as a cost in million dollars ($m) per 100,000 individuals. The (symptomatic) illness attack rate is presented as a % of the population. The percentage reduction from the baseline illness attack rate due to each intervention and the cost (in $) for each symptomatic case averted are also given in Table 3. The strategies which give the lowest range of costs per symptomatic case prevented are those costing less than $1000 (per case averted); these intervention strategies are highlighted in bold in Table 3.

**Table 3:** Simulation and cost analysis results.

Cluster	Intervention strategies	Illness Attack Rate^2^ (S.D.^3^)	Percentage of reduction of baseline Illness Attack Rate due to intervention	Total cost in million $ per 100,000 population	Cost in dollar ($) per case prevented due to intervention
A	Baseline or Unmitigated pandemic	13.01 (0.9)	0	6.26	-
B	ISC^1^ 2 weeks	8.5 (1.1)	34.2	5.9	1308.2
	ISC 4 weeks	8.2 (1.03)	37.2	6.6	1372.1
	ISC 8 weeks	6.8 (0.74)	47.5	11.6	1867.9
	ISC Continuously	3.2 (0.46)	75.4	34.1	3476.1
	ISC 2 weeks + WP^1^ + CCR^1^	7.5 (0.82)	42.1	21	3811.3
C	T^1^	7.6 (1.07)	41.7	6	1109.1
	**T + H** 1	4.6 (0.83)	65.1	5.9	**701.5**
	**T + H + E** 1	3.5 (0.58)	73.2	6.1	**641.4**
**D**	**ISC 2 weeks + T + H**	3.2 (0.57)	75.7	6.2	**632**
	**ISC 2 weeks + T + H + E**	2.8 (0.44)	78.8	6.5	**636.6**
	**ISC 4 weeks + T + H**	3.1 (0.49)	75.8	6.7	**676.1**
	**ISC 4 weeks + T + H + E**	2.7 (0.43)	79	6.6	**640.2**
	**ISC 8 weeks + T + H**	3.1 (0.42)	76.3	7.7	**777**
	**ISC 8 weeks + T + H + E**	2.7 (0.4)	78.8	7.3	**708**
E	ISC 2 weeks + WP + CCR + T	4.1 (0.63)	69.1	22.3	2502.8
	ISC 2 weeks + WP + CCR + T + H	2.8 (0.49)	78.1	21.2	2076.4
	ISC 2 weeks + WP + CCR + T + H + E	2.4 (0.37)	81.5	21.3	2007.5
F	ISC 2 weeks + WP	8.2 (0.97)	37.4	21.1	4386.7
	ISC 2 weeks + CCR	7.5 (1.1)	42.4	5.7	1034.5
	ISC 2 weeks + WP 2 weeks	8.5 (1.1)	34.5	13.6	3015.5
	ISC cont.^1^ + WP cont.	2.6 (0.34)	79.7	103	9894.3

1 Interventions are abbreviated as follows: ISC – individual school closure, T – antiviral treatment, H – household antiviral prophylaxis, E – extended antiviral prophylaxis, WP – 50% workplace closure (4 weeks if no duration is stated), CCR – 50% community contact reduction. “cont.” refers to continuous school or workplace closure.

2 Illness attack rates are presented as a percentage of population.

3 S.D. – Standard Deviation (in % of population) due to 40 simulation realizations for each scenario.

In Figure 2 the illness attack rates for each intervention strategy considered are shown to range from 2.4% (SD – standard deviation - 0.37) to 8.5% (SD 1.1) while that of the unmitigated attack rate is 13% (SD 0.9). The total costs of a particular strategy are superimposed on each attack rate column and indicated by a cross. Figure 2 groups the simulated intervention strategies in six different clusters (from cluster A to cluster F). A captures the single baseline (unmitigated) scenario, cluster B groups individual school closure (ISC) scenarios, cluster C groups solely antiviral drug based scenarios, cluster D groups combined antiviral and limited duration school closure scenarios, cluster E combines social distancing strategies such as workplace non-attendance (WP) and community contact reduction (CCR) with school closure and antiviral drug strategies, cluster F groups certain combinations of school closure and social distancing strategies. We assumed WP (50% reduction in workplace attendance) and CCR (50% reduction in community contact) were effective for 4 weeks unless stated otherwise. In our analysis an intervention strategy is considered cost-effective if it can significantly reduce the illness attack rate with a lower total cost compared to the unmitigated pandemic scenario and to other intervention strategies. Highly cost-effective strategies may be deemed to be those where the cost per symptomatic case prevented are less than a thousand dollars ($1000).

### Baseline, unmitigated pandemic scenario

In the absence of interventions our simulated baseline epidemic had an effective reproduction number R of 1.2 and an illness attack rate of 13% (SD 0.9), closely matching estimates of the H1N1 2009 influenza pandemic [5]. Similar estimates for H1N1 2009 have been found in other settings [4] and coincide with transmissibility estimates for seasonal influenza which uses data from a number of countries [24]. The simulated unmitigated epidemic resulted in a cost of $6.26 million per 100,000 individuals. We report total costs in $m (million dollars) per 100,000 individuals throughout this paper.

### Effective interventions for mitigating a pandemic with H1N1 2009 characteristics

Our results suggest a set of intervention strategies as being highly effective in terms of reducing the attack rate from the unmitigated scenario of 13% (SD 0.9) to between 2.4% (SD 0.37) to 3.5% (SD 0.58). Here the baseline attack rate is reduced by 81.5% to 73.2% respectively. These highly effective interventions are a) continuous school closure (cluster B); b) T+H+E which is the use of antiviral drugs for Treatment and for Household and Extended prophylaxis (cluster C); c) the combination of school closure with antiviral strategies (T+H and T+H+E) (cluster D); d) individual school closure (ISC) for 2 weeks together with workplace and community contact reductions of 50% (WP+CCR) and the T+H+E antiviral strategy (cluster E); e) continuous school closure with continuous WP (cluster F).

We also determined that the total cost of those effective interventions ranged from $6.1m to $103m (see Figure 2 and Table 3). This highlights a significant difference in costs associated with the strategies which are the most effective in reducing the illness attack rate. The lowest attack rate of 2.4% (SD 0.37) (with an 81.5% reduction in cases) resulted from the ISC 2 weeks+WP+CCR+T+H+E strategy (cluster E) which has a total costs of $21.3m ($2007 per case prevented), which is neither the most expensive nor the most cost-effective strategy.

### Cost-effective interventions for mitigating a pandemic with H1N1 2009 characteristics

Antiviral drug strategies combined with limited duration school closure result in attack rates ranging from 2.7% (SD 0.4) to 3.2% (SD 0.57) (see Figure 2, cluster D) compared to the 13% (SD 0.9). For example a strategy of 2 weeks school closure combined with the T+H antiviral strategy costs $6.2m with a resulting attack rate of 3.2%. By contrast, the addition of an extra 6 weeks of school closure used by the ISC 8 weeks+T+H strategy costs $7.7m with a resulting attack rate of 3.1% (SD 0.42).

The use of an Extended antiviral prophylaxis strategy compared to a Treatment and Household only prophylaxis strategy (that is T+H+E compared to T+H) increases costs slightly but reduces the overall attack rate. For example the ISC 2 weeks+T+H+E strategy has costs of $6.5m compared to the cost $6.2m for ISC 2 weeks+T+H with a corresponding reduction in attack rate. Similar patterns are also found for 4 weeks and 8 weeks school closure when combined with the antiviral strategies.

The combination of antiviral drug strategies together with school closure strategies are found to be the most-cost effective (see Table 3, cluster D) in terms of the cost per case prevented, for a pandemic with H1N1 2009 characteristics. Those interventions cost between $632 and $777 per case averted. Closing schools for 2 weeks (ISC 2 weeks) coupled with case treatment with antivirals and prophylaxis for household contacts (T+H) gives the minimum cost of $632 per case prevented, when compared to other strategies. Starting with the ISC 2 weeks+T+H strategy an additional increase in the duration of school closure or an extension of the antiviral prophylaxis regimen to contacts beyond the household (to the T+H+E strategy) each give a limited increases in cost per case averted but are still cost effective (see Table 3, cluster D).

### Impact of antiviral drugs strategies without social distancing

Antiviral drug strategies such as T, T+H and T+H+E result in attack rates of 7.6% (SD 1.07), 4.6% (SD 0.83) and 3.5% (SD 0.58) respectively when compared to the unmitigated attack rate of 13% (SD 0.9). These strategies give a significant reduction in the unmitigated attack rate (in the range of 42% to 73%) with very similar overall costs in the $5.9m to $6.1m range. These costs are lower than the “do nothing” baseline cost of $6.26 m. These antiviral drug strategies are therefore cost-effective with case averted costs of $1109, $701, $641 per individual for the T, T+H and T+H+E respectively (see Table 3, cluster C).

### Impact of duration on the effectiveness and cost of school closure intervention

While school closure only strategies are both less effective (in reducing the attack rate) and less cost effective when compared to combining them with the antiviral strategies (see cluster B compared to cluster D, Figure 2 and Table 3), the duration of school closure is of significance. Increasing the duration of closure increases the effectiveness of the overall attack rate reduction but is increasingly less cost-effective; with per case prevented costs rising from $1308 to $3476 as closure periods increase from 2 weeks to continuously (see Table 3, cluster B).

2 weeks and 4 weeks school closure strategies cost $5.9m ($1308 per case averted) and $6.6m ($1372 per case averted) resulting from attack rates of 8.5% (SD 1.1) and 8.2% (SD 1.03) respectively. Increasing the duration to 8 weeks or continuously, also increases the total costs up to $11.6m ($1868 per case averted) and $34.1m ($3476 per case averted) with attack rates of 6.8% (SD 0.74) and 3.2% (SD 0.46) (see Table 3 and Figure 2, cluster B).

### Impact of social distancing strategies on the effectiveness and cost

Our results indicate that school closure combined with further social distancing measures (reduction in workplace attendance and community contact by 50%) and antiviral drug strategies are effective, giving attack rates of 4.1% (SD 0.63), 2.8% (SD 0.49) and 2.4% (SD 0.37) (see Figure 2 and Table 3, cluster E) but are not cost-effective. The overall cost of these strategies is approximately $22m and the cost per case prevented ranges between $2007 and $2502.

The most costly strategy is that of continuous school closure together with continuous 50% workplace non-attendance (WP) (see Table 3, cluster F). This strategy has the highest overall costs of $103m and the highest case prevented cost ($9894 per case). However it is the second most effective, resulting in the second smallest attack rate of 2.6% (SD 0.34) (a 79.7% reduction in cases from the baseline). In contrast, the most effective strategy, with an attack rate at 2.4% (SD 0.37) involves school closure of 2 weeks coupled with the WP+CCR social distancing and T+H+E antiviral strategies (see cluster E, Table 3). The cost of this strategy is $2007 per case averted in comparison.

### Sensitivity to higher transmissibility

**Figure 3:**
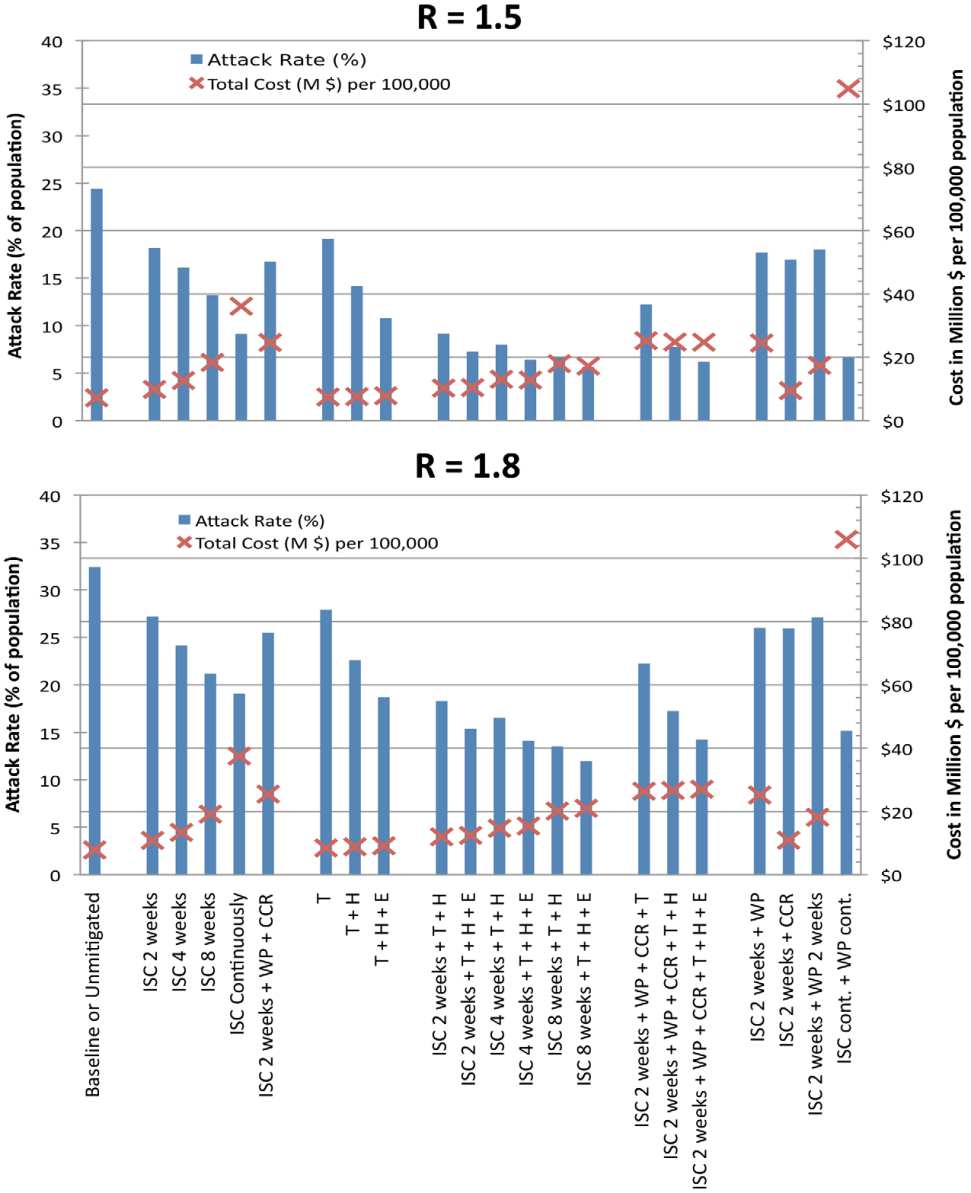
Cost-effectiveness of intervention strategies for higher transmission pandemics. Cost effectiveness for pandemics with higher reproduction numbers (R of 1.5 and 1.8) is shown in terms of illness attack rate (%) and cost in million dollars per 100,000 population. Intervention strategies are abbreviated as follows: ISC – individual school closure, T – antiviral treatment, H – household antiviral prophylaxis, E – extended antiviral prophylaxis, WP – 50% workplace closure (4 weeks if no duration is stated), CCR – 50% community contact reduction. “cont.” refers to continuous school or workplace closure.

Our simulation results and analysis are based on H1N1 2009 pandemic data [5] (attack rate of 13% (SD 0.9) and effective reproduction number R of 1.2) together with health outcome data (such as required GP visits, hospitalisations, ICU treatment and deaths) reflecting the (mild) severity of the pandemic observed in 2009/2010 in Western Australia (unpublished data, Department of Health, Western Australia; personal communication from Dr Gary Dowse). The results are therefore applicable to a future influenza pandemic having severity and transmissibility characteristics similar to that of H1N1 2009. We further extended our simulations and analyses for scenarios with higher transmission characteristics, with effective reproduction numbers R of 1.5 and 1.8, but with the same (mild) severity characteristics, to determine whether increasing the size of the population infected altered the cost-effectiveness results. For both these higher transmissibility scenarios we observed the same cost-effectiveness patterns as those found with an R of 1.2. The attack rate and the total pandemic cost of each intervention scenario for these higher transmission characteristics are shown in Figure 3.

## Discussion

We used a detailed individual-based simulation model to simulate the dynamics of the H1N1 2009 influenza pandemic using 2009/2010 pandemic data. A health-care decision process model has been used to categorise the severity of the pandemic in terms of health outcomes with a costing model used to present the total and per case prevented costs of simulated pandemics under a range of intervention scenarios. These cost outcomes give total costs which include health care cost, the economic cost of lives lost (productivity loss due to death), pharmaceutical costs (antiviral purchase and distribution costs) and productivity loss due to illness and interventions.

The largest contribution to the total cost of the no-intervention strategy was found to be due to productivity losses which arise from illness. This contributes approximately 91% of the total cost in the total pandemic cost (see Table 4, cluster A) compared to other costs (7% and 2% for productivity losses due to death and health care costs respectively).

**Table 4:** Breakdown of costs contributing to total pandemic cost.

Cluster	Intervention strategy^1^	Costs are expressed in million dollars per 100,000 population				
		Health care cost	Productivity loss due to death	Antiviral drugs and distribution cost	Productivity loss due to illness and interventions	Total pandemic cost
**A**	**Baseline or Unmitigated**	0.15	0.40	0.00	5.72	6.26
**B**	**ISC 2 weeks**	0.10	0.26	0.00	5.58	5.94
	**ISC 4 weeks**	0.09	0.25	0.00	6.28	6.62
	**ISC 8 weeks**	0.08	0.20	0.00	11.34	11.62
	**ISC Continuously**	0.03	0.09	0.00	34.06	34.18
	**ISC 2 weeks + WP + CCR**	0.09	0.23	0.00	20.76	21.08
**C**	**T**	0.09	0.23	0.21	5.49	6.02
	**T + H**	0.05	0.14	0.39	5.35	5.93
	**T + H + E**	0.04	0.10	0.69	5.29	6.12
**D**	**ISC 2 weeks + T + H**	0.04	0.09	0.27	5.83	6.23
	**ISC 2 weeks + T + H + E**	0.03	0.08	0.56	5.83	6.51
	**ISC 4 weeks + T + H**	0.04	0.09	0.26	6.34	6.73
	**ISC 4 weeks + T + H + E**	0.03	0.08	0.56	5.97	6.64
	**ISC 8 weeks + T + H**	0.03	0.09	0.26	7.31	7.70
	**ISC 8 weeks + T + H + E**	0.03	0.08	0.56	6.62	7.29
**E**	**ISC 2 weeks + WP + CCR + T**	0.05	0.12	0.11	22.03	22.31
	**ISC 2 weeks + WP + CCR + T + H**	0.03	0.09	0.24	20.84	21.19
	**ISC 2 weeks + WP + CCR + T + H + E**	0.03	0.07	0.50	20.71	21.31
**F**	**ISC 2 weeks + WP**	0.09	0.25	0.00	20.75	21.09
	**ISC 2 weeks + CCR**	0.08	0.23	0.00	5.46	5.77
	**ISC 2 weeks + WP 2 weeks**	0.10	0.26	0.00	13.25	13.61
	**ISC cont. + WP cont.**	0.03	0.07	0.00	102.98	103.08

1 Interventions are abbreviated as follows: ISC – individual school closure, T – antiviral treatment, H – household antiviral prophylaxis, E – extended antiviral prophylaxis, WP – 50% workplace closure (4 weeks if no duration is stated), CCR – 50% community contact reduction. “cont.” refers to continuous school or workplace closure.

### Most effective interventions

We find a set of intervention strategies which give the highest reduction in attack rates compared to the unmitigated situation. These are a) continuous school closure; b) the use of antiviral drugs for Treatment and for Household and Extended prophylaxis; c) the combination of school closure with antiviral strategies; d) individual school closure for 2 weeks together with workplace and community contact reductions of 50% and the T+H+E antiviral strategy; e) continuous school closure with a continuous reduction in workplace contact. However, these strategies give a wide variation in total costs since the highest component of the total cost is productivity loss due to illness and direct productivity loss due to interventions (especially for the workplace non-attendance and continuous school closure strategy). The social distancing measures used in these strategies contribute the greatest component in total costs while all predominantly antiviral drug based strategies are substantially less costly.

### Most cost-effective interventions

A key finding of this study for a future influenza pandemic which has H1N1 2009 characteristics is that antiviral drugs strategies which include treatment and prophylaxis (the T+H and T+H+E strategies) are the most cost-effective, whether used with or without the addition of a limited period of school closure. These strategies give the cost per case prevented in the range of $632 to $777. As the transmission and severity characteristics for H1N1 2009 used in this study relate closely to estimates for seasonal influenza epidemics in the United States, France and Australia [24], these results will also be applicable in a seasonal influenza setting.

We also find that for all cost-effective intervention strategies examined the highest component of the total cost is due to productivity losses from illness and interventions. Losses due to interventions include adults removing themselves from the workplace to perform child care duties following school closure. Compared to health care costs and productivity loss due to death, these productivity losses constitute the highest cost burden (see Table 4, cluster D). The use of antiviral drugs as the sole strategy avoids some of the productivity losses which arise from (some of) these interventions. Pharmaceutical costs and the cost of distribution are less than the productivity losses due to a reduced workforce for child care if school closure interventions are used as an alternative.

The treatment and household prophylaxis antiviral strategy coupled with 2 weeks of school closure has the lowest cost of $632 per case prevented when compared to all other strategies. This cost-effectiveness result aligns with our previous findings that the T+H antiviral strategy combined with 2 weeks school closure has the highest number of prevented cases per antiviral drug course used [7].

Coupling the T+H and T+H+E antiviral strategies with extended school closure (e.g. of 4 weeks and 8 weeks) gives a similar reduction in the attack rate compared to only 2 weeks school closure with the same antiviral strategies, but with an increase in cost per case prevented. This is due to increased productivity losses of working adults required to look after school children during the longer period of school closure.

As a sole intervention measure, all three antiviral drugs strategies (T, T+H and T+H+E) result in small net cost savings compared to the cost of an unmitigated pandemic. Significantly, these antiviral strategies result in substantial illness attack rate reductions of 42% to 73%. This substantial reduction in illness and concomitant death has an overall cost in the $5.9m to $6.1m range (per 100,000 population). These costs are similar to the baseline, no intervention pandemic cost of $6.26m, but prevent significant illness. This result becomes even more significant for a virus strain which is more severe than H1N1 2009 with increased rates of severe illness and death.

Short periods of school closure without other interventions (of 2 weeks and 4 weeks) are only slightly more costly (in term of total costs) than the baseline but give a significant 34% to 37% reduction in cases if optimally timed; the importance of when school closure strategies should be activated to maximize their effectiveness in discussed in [9]). While short-duration school closure is a relatively cost-effective strategy at $1308 and $1372 per case prevented the costs are higher than antiviral-only or combined school closure and antiviral strategies.

The duration of school closure plays an important role in attack rate reduction and in the total cost of a pandemic. Increasing the duration of school closure trades off the overall cost against an increase in attack rate reduction. Increasing the duration can decrease the health care cost and the consequent productivity loss due to death but results in a significantly greater increase in productivity loss due to the interventions. As a result, the continuous closure of schools strategy gives a high $3476 cost per case prevented.

Combining workplace and community-wide contact reductions (WP and CCR) with antiviral drug strategies (that is with T, T+H, T+H+E) and limited period (2 weeks) school closure strategies are highly effective in reducing the attack rate but with a comparatively high cost compared to scenarios which lack this extended, more rigorous social distancing. The health care costs and productivity loss due to death are reduced due to the application of antiviral drugs but the total cost is dominated by the productivity loss arising from the social distancing interventions, especially the workplace reduction strategy. When workplace reduction (either continuously or limited) is coupled with school closure, it causes the greatest increase in the overall pandemic cost though it does give a significant reduction in attack rates. This strategy may be suitable for those countries which have limited access to antiviral drugs or if there is a significant risk of development of antiviral drug resistant influenza strains.

### Related research

There are only a limited number of related studies in the published literature [20], [22], [25]. Two of these [20], [22] use a synthetic, small community-based simulation model somewhat similar to our Albany model while [25] utilises a deterministic, differential equation-based compartmental model. Our model differs from these models in that it was built with the aim of giving us the most faithful replication of the spatial contact structure, mixing groups and community-wide random contacts found it an actual population centre (Albany in Western Australia), given available data sources including detailed census data [26]. To achieve this high level of realism the Albany model therefore encompasses significant complexity.

In the three related studies [20], [22], [25] different assumptions have been made for the scenarios considered, such as pandemic transmissibility (reproduction number and attack rate) and severity (mortality rate) together with intervention strategies which differed from each other and with our study. In the studies reported in [20], [22], [25] the authors simulated influenza pandemics with higher transmissibility and severity characteristics than that which occurred with the H1N1 2009 pandemic and on which our study is based, so making it difficult to directly compare our results.

In [20] the authors simulated a pandemic with an attack rate of 50% (a reproduction number of 2.0) and with severity based on an estimation of the case fatality rate CFR of the 1918 pandemic of 2.5%). This 1918 transmissibility and severity is much higher than those of all subsequent influenza pandemics (1957, 1968, 2009). In [22] the authors simulated pandemics with attack rates of 25% and 35% (and reproduction numbers 1.6 and 2.1) and case fatality rates of 0.25% and 1%. In the third study [25] a reproduction number of 1.7 with an illness attack rate of 31.1% and case fatality rates of 0.75%, 1% and 2% for age groups 0–19, 20–64 and 65+ years respectively was considered. The transmissibility and severity used in the above three studies are significantly higher compared to our H1N1 2009 settings (reproduction number of 1.2, attack rate of 13% and case fatality rate of 0.0045%, which is estimated from [5] and Western Australia Department of Health data (personal communication from Dr Gary Dowse).

While the significant differences in the assumptions used in these studies [20], [22], [25] make direct comparison with our results difficult, we can observe general cost-effectiveness patterns with them. In the two studies reported in [20] and [22] continuous school closure was determined to be the most costly strategy of those considered. When school closure was combined with other strategies (such as antiviral drugs and adult and child contact reduction in the workplace and wider community), the resultant strategies were also shown to be costly. All antiviral drug strategies reported in these two related studies were found to be cost-effective when compared to continuous school closure.

In studies [20], [25] the most cost-effective strategies were determined to be pre-pandemic vaccination coupled with antiviral treatment and prophylaxis, assuming a suitable vaccine is available prior to pandemic onset. In the context of the 2009 pandemic, where a vaccine was not available during the initial phases of the pandemic, study [22] determined the most cost-effective strategy as antiviral treatment and prophylaxis coupled with school closure and a 50% reduction in contact in the workplace and community. These results were obtained using a reproduction number of 2.1 and a CFR of 1%, both of which were much higher than later estimates of the underlying transmissibility and severity of H1N1 2009. They also determined that for a virus strain with lower transmissibility and case fatality rates the school closure component of the above combined strategy causes it to be non cost-effective.

Using the lower transmissibility and severity level found in the 2009 H1N1 2009 pandemic we also found that a continuous school closure strategy was a costly intervention ($34.1m per 100,000), giving the second highest cost among those which we considered. We also simulated the economic impact of a 50% workplace attendance reduction for 4 weeks and this strategy together with continuous school closure gave the highest cost among the simulated scenarios ($103m per 100,000). We found antiviral treatment and prophylaxis strategies were highly cost-effective, a result which confirms those of [20], [22], [25]. We additionally determined that coupling our antiviral drugs strategies with limited duration school closure are the most cost-effective strategies for mitigating a future influenza pandemic having H1N1 2009 characteristics. We also found that the use of limited duration school closure is a significantly more cost-effective strategy when compared to continuous school closure. Strategies for optimising activation timing and the duration of school closure are presented in [9].

We have use US based costs throughout, following the work of Sander et al. [20], which based costs on US fee and price schedule [27], [28], [29]. We considered alternative sources for United Kingdom and Australian productivity and health cost data, sources which were used in analysis presented in [25], [30], [31]. We found that when adjusted for inflation and converted to US dollars these costs were comparable (within 20%) to the costs used in our model. We also considered an alternative source of health care data for the US (the proprietary MarketScan database) used in two previous influenza economic analyses [22], [32], which showed unit health costs 4-6 times higher than the US, UK, and Australian sources mentioned above. However, even these higher health care costs would not have changed the relative outcome of the scenarios presented in this paper. This is because total costs were dominated by the productivity loss due to illness and intervention, with health care costs contributing to only a minor degree. Note that while this is true of the 2009 pandemic, which was relatively mild in terms of health outcomes, the health-care cost component of a pandemic with more severe health outcomes (e.g. with a higher hospitalisation rate) may be significant. In this case any analysis should take into account the ratio of wages to health care costs, which might differ significantly between countries and data sources (particularly the US, which pays significantly more for health care services per capita [33]).

### Policy implications

Our simulation and cost analysis results give guidance to public health policy makers as to the cost-effectiveness of a range of (combined) intervention strategies which may be used during future influenza pandemics with H1N1 2009 characteristics and which are also applicable to seasonal influenza epidemics. From a cost-optimal perspective the use of antiviral drugs are most effective in reducing the cost and attack rates i.e. achieving significant attack rate reductions. To improve effectiveness further a purely antiviral drugs strategy may be combined with limited duration school closure to reduce the attack rate further, but with a slight cost increase. It should be noted that the effectiveness of antiviral drug interventions are dependent on a) the delay occurring between symptom onset and diagnosis and b) the percentage of the infected population being diagnosed; a previous, detailed analysis of these issues is presented in [8]. The use of extreme social distancing e.g. long-term continuous school closure or any form of workplace (WP) reduction (i.e. 50% reduction) is a very costly choice of intervention but may be appropriate if there is limited availability of antiviral drugs or if significant antiviral drug resistance has developed.

As the results of this study are based on a population in a developed country with a westernised health-care system, the outcomes may not be applicable to populations in a developing country, where populations may be less/more mobile and have higher population densities.
